# Engaging Elderly People in Telemedicine Through Gamification

**DOI:** 10.2196/games.4561

**Published:** 2015-12-18

**Authors:** Frederiek de Vette, Monique Tabak, Marit Dekker - van Weering, Miriam Vollenbroek-Hutten

**Affiliations:** ^1^ University of Twente Faculty of Electrical Engineering, Mathematics and Computer Science, Biomedical Signals and Systems, Telemedicine group Enschede Netherlands; ^2^ Roessingh Research and Development Telemedicine Group Enschede Netherlands

**Keywords:** gamification, framework, elderly, older adults, eHealth, telemedicine, adherence, engagement, classification, player type, personality

## Abstract

**Background:**

Telemedicine can alleviate the increasing demand for elderly care caused by the rapidly aging population. However, user adherence to technology in telemedicine interventions is low and decreases over time. Therefore, there is a need for methods to increase adherence, specifically of the elderly user. A strategy that has recently emerged to address this problem is gamification. It is the application of game elements to nongame fields to motivate and increase user activity and retention.

**Objective:**

This research aims to (1) provide an overview of existing theoretical frameworks for gamification and explore methods that specifically target the elderly user and (2) explore user classification theories for tailoring game content to the elderly user. This knowledge will provide a foundation for creating a new framework for applying gamification in telemedicine applications to effectively engage the elderly user by increasing and maintaining adherence.

**Methods:**

We performed a broad Internet search using scientific and nonscientific search engines and included information that described either of the following subjects: the conceptualization of gamification, methods to engage elderly users through gamification, or user classification theories for tailored game content.

**Results:**

Our search showed two main approaches concerning frameworks for gamification: from business practices, which mostly aim for more revenue, emerge an applied approach, while academia frameworks are developed incorporating theories on motivation while often aiming for lasting engagement. The search provided limited information regarding the application of gamification to engage elderly users, and a significant gap in knowledge on the effectiveness of a gamified application in practice. Several approaches for classifying users in general were found, based on archetypes and reasons to play, and we present them along with their corresponding taxonomies. The overview we created indicates great connectivity between these taxonomies.

**Conclusions:**

Gamification frameworks have been developed from different backgrounds—business and academia—but rarely target the elderly user. The effectiveness of user classifications for tailored game content in this context is not yet known. As a next step, we propose the development of a framework based on the hypothesized existence of a relation between preference for game content and personality.

## Introduction

It is expected that 25% of the European population will be older than 65 years in 2050 because of global population aging [1]. Current socioeconomic structures cannot provide enough work force and capital to meet the needs of this rapidly growing elderly population [2]. Telemedicine refers to health services that enable patients to receive treatment in their daily living environment, whereby distance is bridged by information communication technology (ICT) and at least one health care professional is involved, alleviating the increasing demand for elderly care by extending the time of autonomy and independence [3]. Although telemedicine technology seems promising, practical implementation still leaves much to be desired. Several studies have shown that adherence to telemedicine interventions, such as therapy supporting a healthy lifestyle, is low [4] and decreases over time [5], even though these studies showed a significant effect on health outcomes [6]. Clearly, there is a need for strategies that motivate elderly people to use, and keep using, the technologies offered.

Gamification, the application of game elements to nongame fields, may be such a strategy [7]. There is a rapid growth in the number of initiatives that use gamification, illustrating a variety of approaches developed from various viewpoints, including education, behavior change, physical health, and mental health. However, a lack of a refined conceptualization of this strategy exists in these disciplines, and gamification, for elderly people in particular, remains an even further underexplored area. In general, it is not yet known which one of these approaches is the best for the durable engagement necessary for better adherence.

Choice and personalization of content [8], or tailoring, is known to be beneficial for intrinsic motivation [9], which in turn increases long-term engagement needed for adherence. To provide this tailored content, insight is needed into how users should (or want to) be addressed through gamification and how these needs can be classified is required. To our knowledge, information on the practical implementation of existing classifications is not yet available. We believe that once an overview of existing frameworks for gamification and user classification is established, a gamification strategy that is effective in realizing long-term engagement for the elderly user can be developed.

For this purpose, the aim of the paper is to (1) provide an understanding of the theoretical background of gamification, including existing frameworks for developing gamification both in general and specifically for the elderly population, and (2) explore existing user classification theories that may serve for the tailoring of game content to the target user. Because of the newness of this field of research, we opt for a broad view on activities in gamification that occur not only within but also outside of scientific research. In future research, we will work toward a user classification of the elderly population that can be used to develop evidence-based gamification strategies and tangible design guidelines for gamification in health care.

## Methods

In a succession of 3 Internet searches, a broad approach to the subject of gamification was taken to gain insight into the many developments in gamification that occur both inside and outside of the scientific world. We performed a search in the scientific search engines PubMed, Scopus, and Google Scholar and in diverse nonscientific sources: from game designer blogs and conference videos to MOOCs (massive open online courses) and YouTube videos. In this paper, gamification is defined as the use of elements from games in nongame contexts to improve user experience and engagement without making that system a full game as is the case with serious games including exergames (combination of exercise and gaming) [10,11].

First, we have researched the conceptualization of gamification from a theoretical perspective (see Multimedia Appendix 1). Keywords used in combination with gamification were used, including derivatives of these words: “theory,” “definition,” “concept,” “framework,” and “analysis.” In addition, keywords (and derivatives of these) implying practical use were used: “method,” “application,” and “gamify” (singular). Then, a search for gamification combined with “criticism,” “downsides”, and “negative” was performed. Second, we investigated the use of gamification in applications for the elderly population (see Multimedia Appendix 1), entering the following combinations of keywords: “gamification,” “gamif*,” “game,” and “gaming” with “elder*,” “elderly,” “senior,” “old*,” and “aging.” Finally, through the same search method, we have researched user classifications that categorize users by their motivation or stimulant to play in order to gain insight into the user and further determine how to tailor content to the user (see Multimedia Appendix 2). Keywords used were “[user, player, gamer]” combined with “[type, taxonomy, classification, model, style].”

Included in the results were articles and other works that present a theoretical basis for the development of gamification, defined as the presence of a framework that is either theoretical and/or based on established scientific foundations or proven effective through evaluation in practice. Therefore, beyond the scope of our paper are numerous works on gamified applications with a black box design.

## Results

### Gamification Frameworks

This section demonstrates the current state of gamification, starting with the concept of gamification in a broader sense and then focusing on gamification for elderly people. We provide an overview of existing frameworks for gamification along with their contexts and backgrounds. With this, we aim to define the status quo in research and provide a deeper understanding of the concept and its use and misuse.

#### The Conceptualization of Gamification

Gamification has gained popularity in diverse fields such as (interactive) marketing and scientific applications, generating different definitions of gamification. Currently, there is no consensus about a definition, mainly due to the underlying perception of what the game elements are exactly in terms of level of abstraction and whether the gamified application is game-like or not. Gamification is often roughly defined as the use of elements from games in nongame contexts; a more refined definition regards gamification as the identification of that which makes games captivating and engaging followed by the transfer of this knowledge to nongame contexts, increasing user enjoyment [12,13]. While some see gamification as a way to act upon psychological principles as certain game techniques do [14], others define gamification as applying gameful interaction or design with a specific intention without creating a full-fledged game [10] or as the process of improving a service with gameful experiences that support the value creation of the user [15]. In the middle of these definitions, we see gamification as the use of game elements that create a game-like experience in a nongame context without creating a full game.

We found a couple of approaches toward the conceptualization of gamification. One emerges from business practices, such as marketing, customer loyalty, and employee engagement; the other from academia and not sales driven, often specifically aiming to incorporate theories on motivation, engagement, and behavior change. Table 1 illustrates this division of the found articles by author, grouped according to their focus.

**Table 1:** Frameworks for gamification in business and academia.

Business	Academia
Cunningham and Zichermann (2011)	Aparicio et al (2012)
Werbach and Hunter (2012)	Nicholson (2012)
Duggan (Badgeville, 2012)	Sakamoto et al (2012)

In business-oriented, or corporate, gamification, the number of successful initiatives, in terms of increased user engagement or revenue, that use gamification has been rapidly increasing in the past few years [16]. It is estimated that the market spend on gamification solutions will grow exponentially until 2016, and at that time 40% of the world’s top market value companies will be using gamification [17,18]. In gamification for the marketing of consumer products, a well-known success story is that of Nike+ by Nike. This gamified running log app, currently used by 5 million players to track their daily exercise goals, caused revenues in the running category to increase by 30% in 2011 alone [19]. An example of successful enterprise gamification is that of software company SAP. After SAP launched a new, gamified version of their online employee and customer community platform, employee usage increased by 400% and community feedback by 96% [20]. Gamification appears to be more than a fad, illustrated by the existence and ongoing success of companies such as Badgeville [21,22], which provides a platform for gamification of enterprise applications and serves major companies such as Samsung, Deloitte, and Dell [23].

There are several authors within this business orientation, such as Cunningham and Zichermann [12], who provide guidelines for gamification by listing game elements and mechanics such as feedback, achievement, social engagement loops, reinforcement, and status, including practical examples. Werbach and Hunter [14] simplify gamification and consider it a tool for business strategy. Their method offers practical guidelines on how to dissect existing games and use them to gamify other applications. Although this approach lacks intricate game mechanics, gamification is used as a comprehensible tool, presenting game elements as a set of building blocks that, used together, can provide the gamified application.

However, the way gamification is applied in business context receives a lot of criticism as analysts estimate that the bigger part of current gamified applications will not meet their business objectives, mainly due to poor design [24]. Game designers criticize the Cunningham and Zichermann method, stating that the mechanics presented do not contribute to a gameful experience [25,26]. Robertson [25] states that gamification turns into “pointsification” when game elements are simply stripped from games and placed in another application. With this, structural components of games are perceived and used elsewhere to function as core mechanics, ignoring the fact that these mechanics should be the inner workings of games. Bogost criticizes this practice using the term “exploitationware” in an article [26] and blog entry titled “Gamification is Bullshit” [27] and states that gamification disassociates the practice from games created for the sole purpose of making an easy profit. A design may be poor as well when it extensively uses external conditions or reinforcements, as known from operant conditioning [28]. These reinforcements often function as main mechanisms to manipulate behavior and usually present in the form of point and reward systems. A shift from intrinsic to extrinsic motivation can occur through offering external awards, known as the overjustification effect [29], which may lead to an early loss of interest of the user. The initial interest in the (gamified) activity may also disappear once the rewards are no longer, or insufficiently, offered [30], an effect called the “hedonic treadmill” [31]. From this we observe that the development of a good game design concept is often disappearing into the background in corporate gamification initiatives, while it is as essential for creating an engaging experience as it is for traditional games.

Scientific research from within academia, the second approach we distinguish, includes few frameworks on the theoretical foundations of gamification. Aparicio et al [32] developed a framework focusing on intrinsic motivation by incorporating concepts from self-determination theory [33]. According to this theory, intrinsic motivation can increase by satisfying the following psychological factors: competence, autonomy, and relatedness. The framework procedure tells us to (1) identify the main objective, (2) identify which intrinsically motivating factors should be included, (3) determine which game mechanics should be used according to these factors, and (4) evaluate the framework in its final application. Nicholson [34] presents a complex framework for meaningful gamification, integrating user-centered design [35] in combination with self-determination, situated motivational affordance [36], situational relevance [37], and universal design for learning [38]. From these core theories, Nicholson [34] suggests how to provide more intrinsically motivating gamification leading to meaningful engagement. Self-determination can be found along with the transtheoretical model of behavior change [39] in the framework of Sakamoto et al [40], describing a value-based framework. The authors present 5 core values (informative, empathetic, persuasive, economic, and ideological value) that, when used with other game mechanics, can be used to create attractive and intrinsically motivating gamification services.

Several differences between the frameworks from business and academia (Table 2) can be observed. The business frameworks are very concrete; they are simple, provide practical guidelines, and, most importantly, have proven their success in this context. In academia, gamification has not yet reached this state of maturity. The frameworks found on both sides are contradictory: those from academia are conceptual and complex and provide methods that are much more difficult to apply. Therefore, among these are no empirically supported frameworks showing their effectiveness in practice. The frameworks from business are simplified, therefore lacking depth, which may suffice for marketing purposes but possibly not for long-term goals needed for telemedicine applications.

**Table 2:** The contrast between business and academic frameworks.

Business	Academia
Applied	Conceptual
Simplicity	Complexity
Practical guidelines	Methods inexplicit
Proven worthy in practice	Earlier stage of development, less empirical support
Lacking depth, oversimplified	Solid scientific foundation
Short-term engagement suffices	Aiming for durable motivation
Immensely popular	Mostly unknown

#### Gamification for Elderly Users

While gamification is gaining popularity in telemedicine [41], limited information was found on appropriate designs for engaging elderly users. Our search for gamification frameworks did not return any information on how to address the elderly users. We therefore present existing literature that describes explorations of designing gamification for this population group (Table 3). Gerling and Masuch [7] indicate that gamification holds significant potential for elderly users, particularly in gamifying physical and cognitive therapy. The authors state that the main challenge for developing such apps lies within the unfamiliarity of older adults with games, making it difficult to draw content from existing digital games. Link et al [42] face a similar challenge after examining a set of game mechanics (points, status, and badges) and concluding that these have the desired impact on youth but not on older adults.

By contrast, Minge et al [43] see gamification as an opportunity to decrease feelings of fear and frustration that elderly people have toward technology. However, the authors emphasize that success depends on careful design. For example, the study participants did not enjoy aspects of quantification and comparison, which are otherwise very common elements of games.

IJsselsteijn et al [44] also state that digital games hold significant positive potential for elderly users, including therapeutic value and social bonding. Elderly users are underrepresented as consumers of digital games because the games offered are not in line with their accessibility and usability demands or their interests and needs. Design requirements are needed to offer the elderly engaging content. According to IJsselsteijn et al [44], however, no empirical data are available on the categorization of elderly gamers that is necessary to do so, including how this would translate into game content.

**Table 3:** Overview of papers described.

Source	Topic
IJsselsteijn et al (2007) [44]	Design opportunities for engaging games for elderly
Gerling et al (2011) [7]	Potential of gamification for engaging (frail) elderly
Minge et al (2011) [43]	Attitude of elderly toward gamification
Link et al (2014) [42]	Effect of game elements on motivation of elderly

### Classifying Users: Player Taxonomies

**Figure 1:**
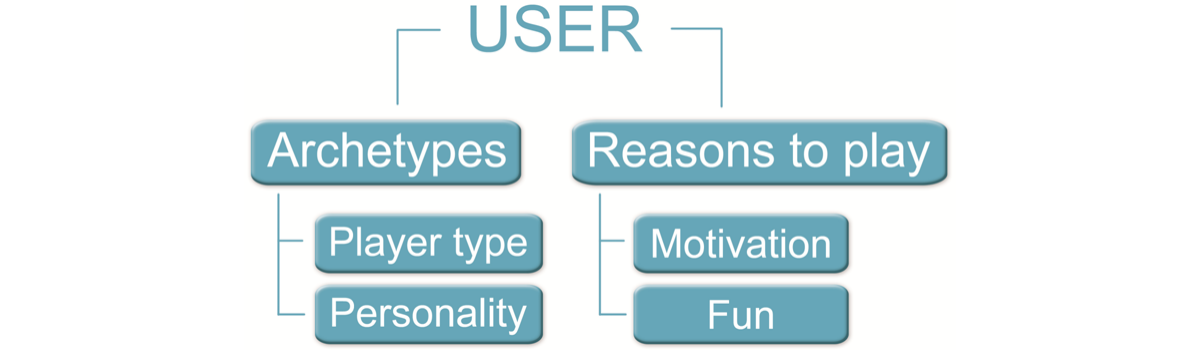
Approaches to classify the user.

User classification holds a key role in the development of tailored game content, as it gives thorough insight into the preferences that individuals or subgroups within a target group may have [45]. However, there are limited valid methods to describe people regarding their gaming preferences [32], and none were found for the elderly user in particular [44]. In this section, we discuss several approaches for classifying users in general, broadly divided into archetypes and reasons to play. Archetypes, player types [46,47] (Bartle, Marczewski), and gaming personality (types) [48] (Vandenberghe) describe the player characteristics while reasons to play, player motivation [49,50] (Yee), and kinds of fun [51,52] (LeBlanc, Lazzaro) take motivating elements as a starting point. In Figure 1, these various approaches are visualized in a diagram. At the end of this section, we summarize and compare these user taxonomies in a chart.

#### Archetypes

**Figure 2:**
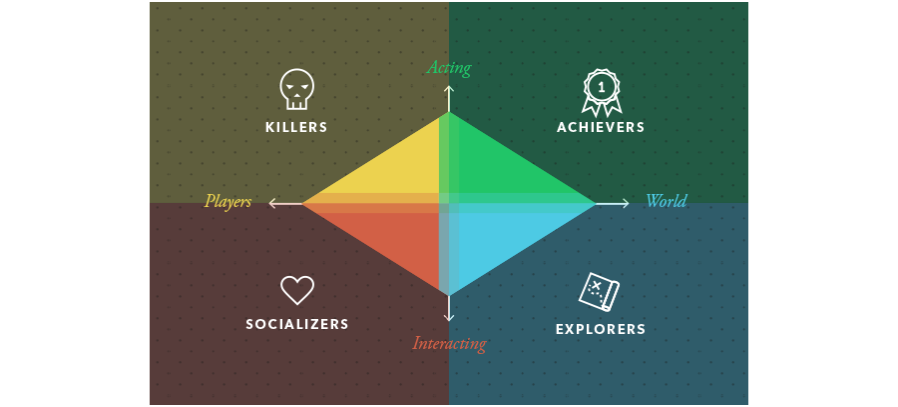
Bartle’s player type model.

The earliest and most cited player taxonomy in a gaming context is the Bartle player type theory. It was developed for the first virtual multiuser environment, text-based dungeons (multiuser dungeons, or MUDs), by observing and analyzing player patterns. Bartle proposes 4 player types (Figure 2) based on two primary interests in gameplay: between the emphasis on players or on the environment and between acting (to) and interacting (with). Achievers are interested in actions on the world and find mastery of the game and competition most compelling; explorers like to interact with the world and enjoy discovery. Socializers are most interested in interacting with other players and enjoy the game for friendships and contacts, while killers are interested in acting on other players, demonstrating their superiority. According to Bartle, a good MUD contains the 4 player types in equilibrium [46]—not necessarily of equal number—and the player types were created to balance the design of these multiplayer games to accommodate for all player types’ play style. The application of this model outside its context is something Bartle himself advises against [45], especially for use in gamification. Furthermore, this model has been criticized for lacking proper validation with empirical data and means to assess players to a type [53,49] and for missing similarity between the virtual world of the MUD and the gamified application. Bartle suggests that the types are exclusive but, in practice, they can be overlapping or mixing [12].

**Figure 3:**
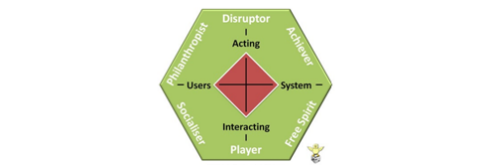
Marczewski’s player type model.

Similarly, but in the context of enterprise gamification, Marczewski [54] proposes a conceptual taxonomy choosing intrinsic motivations from different theories—autonomy; purpose and mastery; change—and the extrinsic motivation, rewards. This results in 6 player types (Figure 3). The axes are equal to the Bartle model but replace player for user and world for system.

Another approach to create player archetypes is through personality. Personality traits have been extensively studied and researched since the 1880s [55] and, although thousands of traits can be found to describe personality [56], a statistical factor analysis demonstrated 5 main factors that many psychologists believe are sufficient [57,58]. The five-factor model (FFM), or Big Five, is currently the most popular and has shown to be reputable, predictive (even normally distributed), reliable, crossculturally tested, and universal [59-63].

In the context of games and gaming, several attempts on predicting the effectiveness of the application of FFM showed inconsistent results [64,65]. In one study, personality traits have been related to preference for game genres [66]. A low predictive capability was found, which may be caused by a lack of evidence on whether the FFM is a valid method to measure personality in a game or not [67,68]; however, direct correlations between the FFM and gaming were researched and described by Vandenberghe [48]. He states that personality is very accurately predictive of gaming preferences and that people play with the same motivations they have in real life or look to express a particular part of personality that is unsatisfied in real life. In his model, the 5 domains of play, a translation of the original FFM traits is made into aspects of gaming motivation (Table 4). Each player is ranked on a linear scale on each of the 5 domains, thereby creating a character description rather than a categorization into a single player type. At the same time, the domains provide insight into the type of content that satisfies the player.

**Table 4:** Five-factor model traits and corresponding gaming motivation traits (deduced from Vandenberghe [48]).

Low score	Trait	High score
Cautious, predictable	Openness to experience	Inventive, curious
*Repeating, conventional*	*Novelty*	*Open, imaginative experiences*
Careless, impulsive	Conscientiousness	Efficient, organized
*Low effort and self-control*	*Challenge*	*High effort and self-control*
Reserved, solitary	Extraversion	Energetic, outgoing
*Relaxing, low social engagement*	*Stimulation*	*Exciting, high social engagement*
Analytical, detached	Agreeableness	Friendly, compassionate
*Competition, defeating*	*Harmony*	*Cooperation, helping*
Confident, secure	Neuroticism	Nervous, sensitive
*Cheerful, comforting*	*Threat*	*Gloom, horror, high tension*

Two examples illustrate specific gaming elements derived from motivation facets. First, the imagination of the user correlates with a preference for either fantasy or realism: someone who scores high on imagination will tend to prefer games that take place in exotic worlds, whereas someone with a low score will prefer games that take place in a world much like ours. Second, scoring high on adventurousness correlates with a preference for exploration and a desire for encountering new things, much like the Bartle type explorer, whereas a low score indicates a preference for local play styles such as building or farming that do not involve leaving the boundary of the known [69].

#### Reasons to Play

**Figure 4:**
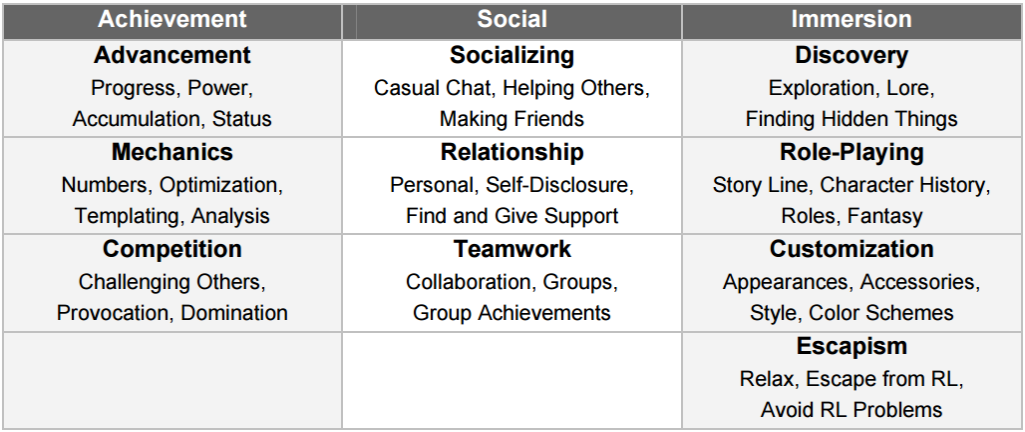
Yee’s model motivations of play in MMORPGs: the components and subcomponents.

Yee [70] proposes a taxonomy based on users’ reasons to play and used a long-term, qualitative analysis and factor analytical approach to create a taxonomy based on player motivations in MMORPGs (massive multiplayer online role-play games). The model by Yee consists of 10 subcomponents factored into 3 main components with which they are most correlated (Figure 4). Each subcomponent is linked to game elements from which players derive satisfaction. He finds that the killer must be omitted and merged into his component of achievement and the original explorer type must be divided into mechanics and discovery. The Yee model is similar to Bartle’s but overcomes several of its weaknesses. For example, the components of Bartle types are not highly correlated, the types overlap and are not distinctive, and a practical way to assess users is lacking. However, similar to the Bartle typology is its narrow focus on massive online gaming.

A taxonomy of game aesthetics, or what makes a game fun, can be found in the mechanics, dynamics, and aesthetics framework by LeBlanc et al [51]. As much as 8 kinds of fun are defined: sensation, fantasy, narrative, challenge, fellowship, discovery, expression, and submission. These aesthetics are used to describe why certain players engage with certain games and more regard the game than categorize the player. Similarly, also focusing on fun as a reason to play, Lazzaro [52] conducted a study to clarify how to address emotions in games without using a storyline by learning what (adult) players found were good gaming experiences. The “4 keys” to fun are

- *Hard fun:* players like challenge, strategy, problem solving, experiencing frustration.
- *Easy fun:* players like intrigue and curiosity and enjoy immersion.
- *Altered states:* players search for internal sensations such as excitement.
- *The people factor:* players use games for social experiences.

#### Overview of Taxonomies

**Figure 5:**
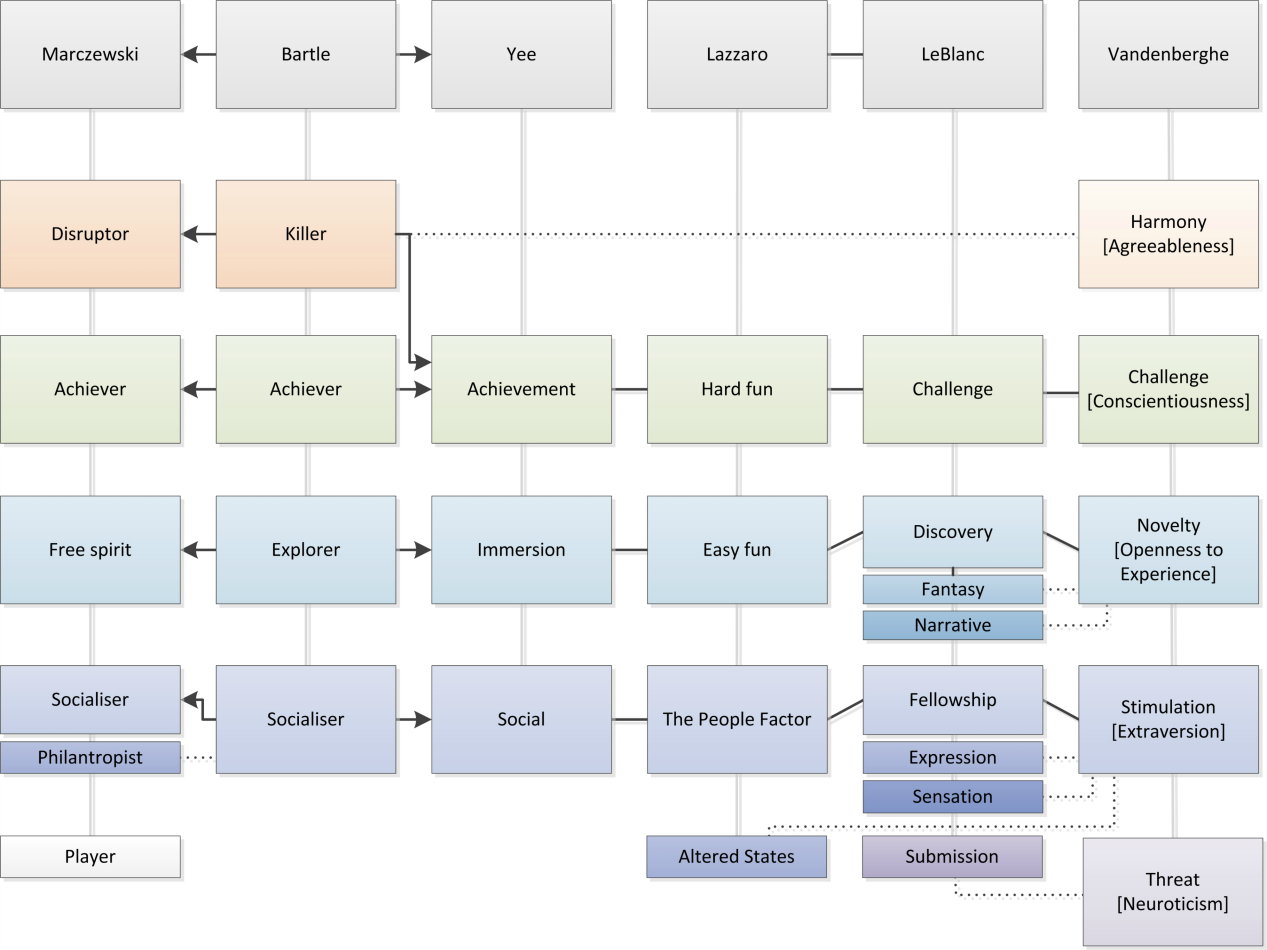
Chart of connections between taxonomies (arrow: direct derivative of, line: high similarity in concept, dots: closely related concepts).

Although the taxonomies aforementioned appear very different concerning the types of classes, many parallels can be found between the characteristics of each class. We present the results in an overview chart (Figure 5). The top row in gray shows the author of the model, and under each author the defined classes (types, motivations, facets, etc) are shown. Arrows indicate a direct derivative of a model, as explained in the previous section; black lines indicate which classes show highly similar characteristics. The dotted line indicates that classes only have several characteristics in common. The colors indicate which classes belong to the same group. This overview shows that there is great connectivity between the models and highlights that the model of Vandenberghe covers all class properties of the other models (except for the player in the Marczewski model).

In the models of Marczewski and Yee, which both have Bartle as point of reference, we see a clear analogy between the achievers and socializers and also in the attributes of the free spirit (interacting with the system, autonomy), the explorer (interacting with the world), and immersion (discovery, exploration). Although Yee does not have a separate type for the killer or disruptor, provocation and domination are present in achievement. Linking to Lazzaro and LeBlanc, achievement is similar to the concept of hard fun and challenge; easy fun (which includes the motive of immersion) and discovery are similar to exploring; and the people factor and fellowship and expression relate to the social aspect. The model of Vandenberghe not only seems all-embracing, but it also adds a dimension to each personality trait. The killer can be linked to a very low score on harmony, the achiever to a high score on challenge, the explorer to a high score on novelty, the socializer to a high score on stimulation. The trait threat is quite unique and only linked to submission. According to Vandenberghe, this trait may not be pointing out what keeps a player playing but what makes the player decide to stop playing.

None of the taxonomies presented target the elderly user specifically. Furthermore, we do not know of any methods regarding the mapping of this target group on the existing taxonomies, mainly because the gaming industry does not focus on this group as a consumer for video games. Moreover, the taxonomies are in most cases designed for use in a specific application, such as enterprise gamification or MMORPGs, and it is not known how suitable they are for application in telemedicine interventions. We can identify many parallels between the models, and we consider that the 5 domains of play stand out from the rest. Unlike the other models, an individual is not given a singular class label or a combination of those. Instead, a complete character description can be created based on preference for certain aspects or elements of games. What makes this theory even more attractive is that it describes the user based on personality, a universal understanding regardless of age.

## Discussion

### Principal Findings

The first objective of this study was to provide an overview of theoretical frameworks for the application of gamification and of methods for gamification that specifically target the elderly user. Second, we have explored user classification theories, which are needed to gain insight into the user and serve as a tool to effectively tailor content. We have found that current frameworks for gamification rarely target the elderly user. The effectiveness of the use of user classifications for tailored game content is not yet known, neither are there indications for classifying the elderly user with these theories. How can we use these results to systematically design effective gamified telemedicine applications for elderly?

Frameworks for gamification emerge from two main approaches. First, there is a business-oriented approach, with examples of success in practice, using an easy-to-apply framework to gamify applications. However, the frameworks from this approach may also be oversimplified, which suffices for marketing purposes but possibly not for long-term engagement needed in telemedicine. Second, frameworks created within academia target for higher causes, such as better education and health outcomes. These frameworks often make use of established theories but are complex, and, at the time of writing, not used in practice. In both approaches, no appropriate framework was found to design gamification for elderly users and application in telemedicine. Therefore, a new framework should be created that is of sufficient depth but applicable in practice and supported by empirical data on its effectiveness. To do so, we would position our future research in academia and take example of the studies presented within this approach. Just like the authors discussed [32,34,40], we would aim for qualitative, long-term engagement and focus on stimulating intrinsic motivation.

Our study showed two approaches for user classification theories: archetypes, where classes are user types with associated preferences, and reasons to play, where classes are based on attributes that describe the user preference. None of the found taxonomies seem to be applicable in telemedicine for elderly users due to the very different context and audience for which they have been developed and the fact that we are not familiar with the use of these taxonomies in practice. However, a high level of understanding of the target group will greatly contribute to designing effectively engaging content. This can be achieved by a taxonomy for game design specifically for elderly users. Creating such a taxonomy and corresponding game content can be difficult, because older adults may relate to video games differently than younger users as they might not be able to draw from earlier experience with video games. To create such a classification, it would be most desirable to observe the behavior of intended users in games, but the scarcity of elderly gamers (and limited availability of games for elderly people) does not provide sufficiently representative subjects for the whole target group.

Although from the taxonomies found none seem directly suitable for creating our future framework, the 5 domains of the play model [48] exceed the stereotypical classes of the other models by providing a detailed insight and overview of motivations users may have. The model provides an overview of both player and preferences (where others use, for example, game genres, which are ambiguous, not clearly outlined, and differing for each producer of video games) and is moreover based on a universally applicable psychological concept that may help in overcoming the particular challenge of mapping a group of users onto a taxonomy who have not been exposed to games at a young age. Therefore, we believe the model by Vandenberghe advances on earlier classifications, thus making it unique and worthwhile to explore further for use in game design for elderly users.

Advantages of creating a framework within the academic approach are the possibility of using solid scientifically established theories and incorporating existing motivational theories and instruments that relate to the objective of gamification to motivate and engage. Serious games and exergames for elderly users [71,72] were not included in our study because our present focus is on improving adherence to existing health interventions by means of gamification, and serious games are full games that require a different approach. However, gamification in persuasive (game) design [73-75] or vice versa and gamification for behavior change [76] [77] deserve to be explored. Furthermore, because a well-designed game concept is essential for creating a motivating experience for the user, relevant game design principles that consider the aspect of experience on engagement such as flow [78,79], immersion [50], and customization [8] can prove useful in reaching our goals. Furthermore, we emphasize the necessity of a good game design concept to successfully gamify an application for engagement. The framework we aim to develop in the future should always leave room for the creative process that is involved. We may be able to predict the preference of a user for different types of content but how content is then designed according to these preferences to appeal to the player could be more art than science.

### Conclusion

We suggest developing a framework for gamification that is based on solid scientific foundations and includes a user classification that specifically assesses the elderly user. We base this classification on the 5 domains of the play model that predicts the existence of a relation between preference for game content and personality. In a study, we need to explore this relation as well as opportunities for use for the intended target group and context. When we know more of these aspects, a gamification framework can be developed by which the classification of the elderly user is used to effectively create tailored, engaging game content. Subsequently, the framework needs to be put to practice and evaluated for empirical support of its effectiveness.

## Appendix

Multimedia Appendix 1 Keywords first search.

Multimedia Appendix 2 Keywords second search.

## Abbreviations

FFM: five-factor model
ICT: information communication technology
MMORPG: massive multiplayer online role-play games
MOOC: massive open online course
MUD: multiuser dungeon
