# Supplementary material for: Engaging Elderly People in Telemedicine Through Gamification
Source: JMIR Serious Games. 2015 Dec 18;3(2):e9. doi: 10.2196/games.4561 (PMC4704903; doi:10.2196/games.4561)
Supplement: Multimedia Appendix 1 [file games_v3i2e9_app1.pdf]

Multimedia Appendix 1. Keywords first search.

gamification	theory	method	criticism	elderly
gamif*	definition	application	downsides	senior
game	concept		negative	old*
gaming	framework			aging
	analysis			
