# Supplementary material for: Engaging Elderly People in Telemedicine Through Gamification
Source: JMIR Serious Games. 2015 Dec 18;3(2):e9. doi: 10.2196/games.4561 (PMC4704903; doi:10.2196/games.4561)
Supplement: Multimedia Appendix 2 [file games_v3i2e9_app2.pdf]

Multimedia Appendix 2. Keywords second search.

user	type
player	taxonomy
gamer	classification
	model
	theory
	style
